# Cognitive function and number of teeth in a community-dwelling population in Japan

**DOI:** 10.1186/1744-859X-12-20

**Published:** 2013-06-24

**Authors:** Yuki Saito, Norio Sugawara, Norio Yasui-Furukori, Ippei Takahashi, Shigeyuki Nakaji, Hiroto Kimura

**Affiliations:** 1 Department of Oral and Maxillofacial Surgery, Hirosaki University School of Medicine, Hirosaki 036-8562, Japan; 2 Department of Neuropsychiatry, Hirosaki University School of Medicine, Hirosaki 036-8562, Japan; 3 Department of Social Medicine, Hirosaki University School of Medicine, Hirosaki 036-8562, Japan

## Abstract

**Background:**

It has been reported that oral health is poor in elderly populations and is associated with poor cognition and dementia. The objective of this study was to examine the association between tooth loss and cognitive function in a community-dwelling population in Japan.

**Methods:**

We examined the association between tooth loss and cognitive function in 462 Japanese community-dwelling individuals. The Mini-Mental State Examination (MMSE) was employed to measure global cognitive status. A multiple logistic regression analysis, with both crude and adjusted conditions for confounding factors, was used to assess the relationship between poor cognition and the number of remaining teeth.

**Results:**

The overall prevalence of poor cognition (MMSE ≤ 23) in this study population was 5.6%. Subjects with poor cognition were significantly older, less educated, scored lower in intellectual activity, and had fewer remaining teeth than those with normal cognition. According to the multiple logistic regression analysis, a lower number of teeth (0–10) was found to be a significant independent risk factor (OR = 20.21, 95% confidence interval = 2.20 to 185.47) of cognitive impairment.

**Conclusions:**

This cross-sectional study on a Japanese community-dwelling population revealed relationships between tooth loss and cognitive function. However, the interpretation of our results was hampered by a lack of data, including socioeconomic status and longitudinal observations. Future research exploring tooth loss and cognitive function is warranted.

## Background

Age-related cognitive impairment is an early sign of clinical dementia and has been reported to contribute to disability [1], morbidity [2], and mortality [3]. Recent community-based surveys in Japan showed that the prevalence of mild cognitive impairment and dementia were 23.4% and 16.4%, respectively [2]. The identification of clinical markers predicting cognitive impairment in elderly individuals is often considered useful in easing the public health burden of poor cognition.

Many factors may contribute to cognitive impairment in older adults, including hypertension, cardiovascular disease, diabetes, dyslipidemia, and lifestyle factors such as smoking, alcohol, and diet [4]. In addition, education, exercise, and active social engagement may be protective factors [4]. However, the abovementioned factors are interrelated in a complex manner that is not completely understood.

Tooth loss has been implicated to be associated with poor cognitive function in some studies of elderly populations. Using data from a cross-sectional Health Survey for England, Stewart et al. found that tooth loss was significantly associated with cognitive impairment even after adjusting for covariates in a community population [5]. They also showed that 40.4% of the community samples and 67.9% of the home care samples were edentulous. In addition, the Nun Study from the USA showed that patients with a low number of teeth had an increased risk of dementia based on longitudinal dental records [6].

The aim of the present study was to determine whether there is an association between tooth loss and cognitive function. For the evaluation of cognitive function, we employed the Mini-Mental State Examination (MMSE). Furthermore, we investigated the risk factors for tooth loss in a community-dwelling population in Japan.

## Methods

### Participants

The subjects consisted of 462 volunteers (163 males and 299 females, all 60 years old or over) who participated in the Iwaki Health Promotion Project in 2012. The data collection for this study was approved by the Ethics Committee of Hirosaki University School of Medicine, and all subjects had provided written informed consent before participating in the study. The demographic (age, gender, education level) and lifestyle (smoking, drinking) data and medical history were obtained from self-report questionnaires and interviews.

### Procedure

A dental examination was performed by two dentists under artificial lighting, with both the dentist and the subject in a seated position. The teeth present were defined as healthy, carious, or treated.

The MMSE was administered to all participants to measure their global cognitive status. This test assesses orientation to place and time, short-term memory, episodic long-term memory, subtraction ability, ability to construct a sentence, and oral language ability. The maximum score was set at 30, and poor cognition was defined as a score less than 23 [7].

The ability to perform activities of daily living was evaluated using the Tokyo Metropolitan Institute of Gerontology Index of Competence (TMIG-IC), a multidimensional 13-item scale [8]. The TMIG-IC was developed to measure the functional capacity of an independently living elderly and is composed of three competencies: instrumental self-maintenance (five items), intellectual activity (four items), and social role (four items). The TMIG-IC was used in previous studies to examine the functional capacity of community-dwelling Japanese elderly [9].

The Japanese version of the Center for Epidemiologic Studies Depression Scale (CES-D) [10,11] was administered to all of the participants to measure their depression symptoms. The CES-D is a 20-item self-report measure that focuses on depression symptoms during the week prior to administering the questionnaire. The maximum score on this scale is 60, and depression was considered to be present when a subject had a CES-D score of 16 or more.

### Statistical analysis

Student's unpaired *t* test (for continuous variables) or the chi-square test (for categorical variables) was used to compare subjects with and without poor cognition. A logistic regression analysis with both crude and adjusted conditions for confounding factors (age, gender, level of education, smoking status, habitual alcohol intake, positive history of diabetes mellitus, hypertension, cancer, TMIG-IC score, and CES-D total score) was used to assess the relationship between poor cognition and the number of remaining teeth. Additionally, a multiple linear regression analysis was applied to determine the factors associated with the number of teeth lost in a community-dwelling population. A *p* value <0.05 was considered to be statistically significant. The data were analyzed using the PASW Statistics PC software for Windows, version 18.0.0 (SPSS Inc., Chicago, IL, USA).

## Results

### Sample characteristics

The subjects were divided into two groups according to their MMSE scores (poor cognition, MMSE ≤ 23, *n* = 26; control, MMSE ≥ 24, *n* = 436). The clinical characteristics of the subjects are listed in Table 1. The subjects with poor cognition were significantly older and less educated, and they had lower TMIG-IC scores, fewer remaining teeth, and a higher amount of teeth lost compared with the control group. No other differences were observed in all of the other characteristics.

**Table 1:** Demographic characteristics of the subjects

	**Poor cognition (*n *= 26)**	**Control (*n *= 436)**
MMSE*	21.0 ± 2.0	28.6 ± 1.7
Age (years)*	73.7 ± 7.9	68.3 ± 6.2
Gender	Male 12, female 14	Male 151, female 285
Level of education (years)*	8.7 ± 1.5	10.8 ± 1.9
Smoking status		
No	18	339
Current	2	31
Previous	6	66
Habitual alcohol intake		
No	14	273
Current	11	149
Previous	1	14
Positive history of		
Diabetes mellitus	3 (11.5%)	44 (10.1%)
Hypertension	13 (50.0%)	201 (46.1%)
Cancer	3 (11.5%)	34 (7.8%)
TMIG-IC score		
Instrumental self-maintenance	4.6 ± 0.8	4.9 ± 0.3
Intellectual activity*	2.9 ± 1.1	3.6 ± 0.7
Social role	3.4 ± 1.0	3.7 ± 0.6
CES-D total score	11.9 ± 7.1	10.3 ± 5.9
Number of remaining teeth*	5.8 ± 7.3	16.2 ± 9.7
Number of teeth lost*	22.3 ± 7.1	11.9 ± 9.6

Student's unpaired *t* test for continuous variables or chi-square test for categorical variables was used to evaluate the differences between poor cognition (MMSE ≤ 23) and control (MMSE ≥ 24). Values for the different tests are presented as mean ± S.D. *MMSE* Mini-Mental State Examination, *TMIG-IC* Tokyo Metropolitan Institute of Gerontology Index of Competence, *CES-D* Center for Epidemiologic Studies for Depression scale. *p < 0.05.

### Relationship between the number of remaining teeth and cognitive decline

Table 2 presents the results of the logistic regression analysis used to assess the relationship between the number of remaining teeth and cognitive decline. After adjusting for confounding factors, 0–10 remaining teeth (OR = 20.21, 95% confidence interval (CI) = 2.20 to 185.47) was shown to be an independent risk factor for cognitive decline. Under both crude and adjusted conditions, a higher level of education and a higher intellectual activity score (TMIG-IC) were consistently associated with a lower risk of cognitive impairment.

**Table 2:** **Risk factors associated with having lower MMSE scores (**≤**23) as estimated by logistic regression analysis**

**Parameter**	**Crude odds ratio (mean (95% CI))**	***p *value**	**Adjusted odds ratio (mean (95% CI))**	***p *value**
Age (years)	1.12 (1.06–1.19)	<0.001	0.99 (0.91–1.08)	0.847
Male	1.62 (0.73–3.59)	0.236	2.98 (0.71–12.53)	0.136
Level of education (years)	0.53 (0.41–0.68)	<0.001	0.52 (0.35–0.78)	<0.01
Smoking status				
No	1.00		1.00	
Current	1.22 (0.27–5.48)	0.800	0.88 (0.11–6.81)	0.904
Previous	1.71 (0.66–4.48)	0.273	1.89 (0.44–8.20)	0.395
Habitual alcohol intake				
No	1.00		1.00	
Current	1.44 (0.64–3.25)	0.381	0.75 (0.19–2.98)	0.683
Previous	1.39 (0.17–11.36)	0.757	1.08 (0.07–15.81)	0.954
Positive history of				
Diabetes mellitus	1.16 (0.34–4.03)	0.813	0.36 (0.06–2.14)	0.259
Hypertension	1.17 (0.53–2.58)	0.699	0.73 (0.23–2.28)	0.589
Cancer	1.54 (0.44–5.40)	0.498	1.74 (0.35–8.73)	0.503
TMIG-IC score				
Instrumental self-maintenance	0.35 (0.18–0.69)	<0.01	0.58 (0.20–1.75)	0.336
Intellectual activity	0.38 (0.25–0.57)	<0.001	0.57 (0.33–0.98)	<0.05
Social role	0.60 (0.37–0.99)	<0.05	0.55 (0.29–1.06)	0.074
CES-D total score	1.04 (0.98–1.11)	0.198	0.98 (0.90–1.08)	0.723
Number of remaining teeth				
22–32	1.00		1.00	
11–21	7.34 (0.85–63.57)	0.070	3.50 (0.32–38.25)	0.305
0–10	27.33 (3.62–206.21)	<0.01	20.21 (2.20–185.47)	<0.01

### Factors associated with the number of teeth lost

The results of a multiple linear regression analysis that included age, gender, education level, smoking status, and habitual alcohol intake; positive history of diabetes mellitus, hypertension, and cancer; and TMIG-IC score, CES-D total score, and MMSE total score are shown in Table 3. Age, education level, current smoking status, positive history of diabetes mellitus, and MMSE total score were independently and significantly associated with the number of remaining teeth.

**Table 3:** Multiple regression analysis of tooth loss

**Parameter**	**Partial regression coefficient**	**Standard error**	***t *value**	**Beta**	***p *value**
Age (years)	0.476	0.075	0.311	6.375	<0.001
Male	−0.873	1.188	−0.043	−0.734	0.463
Level of education (years)	−0.787	0.245	−0.158	−3.217	<0.01
Smoking status					
Current	4.657	1.746	0.123	2.667	<0.01
Previous	2.259	1.320	0.084	1.712	0.088
Habitual alcohol intake					
Current	−1.728	1.126	−0.084	−1.535	0.126
Previous	−1.883	2.462	−0.034	−0.765	0.445
Positive history of					
Diabetes mellitus	3.100	1.398	0.095	2.218	<0.05
Hypertension	0.412	0.885	0.021	0.466	0.642
Cancer	1.994	1.514	0.056	1.317	0.189
TMIG-IC score					
Instrumental self-maintenance	1.863	1.245	0.067	1.497	0.135
Intellectual activity	−0.679	0.653	−0.049	−1.041	0.299
Social role	0.573	0.658	0.038	0.870	0.385
CES-D total score	0.073	0.070	0.044	1.035	0.301
MMSE total score	−0.490	0.189	−0.124	−2.592	<0.05

## Discussion

This cross-sectional study was designed to evaluate the relationship between tooth loss and cognitive function in a community-dwelling Japanese population. Severe tooth loss (0–10 teeth remaining) was found to be significantly associated with poor cognitive function after adjusting for confounders. Furthermore, the number of teeth lost was significantly correlated with age, education level, current smoking status, positive history of diabetes, and MMSE total score in a community-dwelling population in Japan.

To date, some studies have shown a relationship between tooth loss and cognitive function in community-dwelling elderly individuals. In a study on a Swedish population of individuals aged 35 to 85 years [12], edentulous individuals showed lower MMSE scores than those with natural teeth. Even after matching for gender, age, social variables, diseases, stress, and MMSE scores, the cognitive disadvantage of the edentulous group was still apparent. The national Finnish data on individuals aged 55 years and older [13] also showed that individuals with low MMSE scores (0–9) often have no teeth or dentures and less frequently have good denture hygiene than those with high MMSE scores (12–16). In addition, a population-based study on individuals aged 60 to 79 years in Germany [14] reported a significant association between low MMSE scores and the number of remaining teeth after excluding subjects with a history of stroke, traumatic brain injury, Parkinson's disease, multiple sclerosis, or epilepsy.

Several explanations have been hypothesized for the association between poor cognitive function and tooth loss. Periodontal disease, a chronic infection caused by bacteria in the tissue surrounding the teeth, is a common cause of tooth loss in the elderly [15]. The localized inflammatory reaction associated with periodontal disease may lead directly or indirectly to a state of chronic low-grade systemic inflammation associated with raised levels of cytokines [16,17]. Periodontal disease may contribute to cognitive impairment through systemic inflammation [18,19]. Another explanation is that a reduced number of teeth may be associated with nutritional deficits, especially in relation to B vitamins [20,21], which may also play a role in cognitive decline. A third explanation is that the association observed in this study may be due to some unmeasured or unobserved confounding factor. Vascular risk factors [22] and low socioeconomic status [23] may have played a role in the development of cognitive decline. However, assessment of the abovementioned factors was limited in this study, and we cannot rule out the effects of underlying factors. Finally, reversed causality may also be an explanation for our results. Individuals with poor cognitive function may be more likely to have (or to develop) poor oral health because they have a lower ability to perform proper tooth brushing, manage dentures, and use dental-related medications [24].

In this study, we found a relationship between the number of teeth lost and diabetes. Previous studies have shown a relationship between diabetes and tooth loss [25,26]. Long-standing hyperglycemia could lead to the formation of advanced glycation end products (AGEs) [27]. AGEs can transform macrophages into cells with a destructive phenotype, leading to the production of high levels of cytokines and increasing the permeability of the endothelium [28]. Patients with diabetes have these predispositions, which could cause infections and an impaired healing process.

We also found an association between smoking and the number of teeth lost. Substances in tobacco smoke can destroy the supporting tissue of the teeth by causing the dysfunction of gingival fibroblasts, a decrease in microcirculatory function, and immune system deficiency [29-31]. In addition, an impaired ability to repair damaged tissue may aggravate the periodontal destruction in smokers.

There are several limitations to our study. First, the cross-sectional nature of the study does not allow for causal assumptions regarding the relationship between tooth loss and poor cognitive function. Future studies with longitudinal designs are needed to investigate these associations. Second, we administered only the MMSE for the assessment of cognitive function. Although the MMSE is suitable for the screening of some cognitive functions, including orientation to place and time, short-term memory, episodic long-term memory, subtraction ability, and attention, the MMSE score does not always reflect the cognitive function exactly; it is known to be influenced by the education level of the subject. Third, several potential confounding factors, such as socioeconomic level and denture usage, were not assessed in our study. Fourth, as all of the participants were volunteers who were interested in their health, they may have been healthier than the general population. Thus, the cognitive function and tooth number of members of the community who were not in the study may vary from those of the study participants. This ‘selection bias’ must also be considered in studies on community populations. Finally, as our sample size was relatively small, we cannot completely rule out beta error as the cause of our failure to detect associations between tooth loss and cognitive function.

## Conclusions

This cross-sectional study on a Japanese community-dwelling population revealed relationships between tooth loss and cognitive function. However, the interpretation of our results was hampered by a lack of data, including socioeconomic status and longitudinal observations. Therefore, future research exploring tooth loss and cognitive function is warranted.

## Competing interests

The authors declare that they have no competing interests.

## Authors’ contributions

YS conceived of the study and wrote the initial draft of the manuscript. NS designed the study, conducted the statistical analyses, interpreted the data, and assisted in drafting the manuscript. NYF contributed to the study design and interpretation of the results and had full access to all of the data in the study. IT and SN participated in the data collection. HK took responsibility for the integrity of the data and the accuracy of the data analysis. All authors read and approved the final manuscript.
